# Hypertrophic cardiomyopathy and ultra-endurance running - two incompatible entities?

**DOI:** 10.1186/1532-429X-13-77

**Published:** 2011-11-29

**Authors:** Mathew G Wilson, Navin Chandra, Michael Papadakis, Rory O'Hanlon, Sanjay K Prasad, Sanjay Sharma

**Affiliations:** 1 ASPETAR, Qatar Orthopaedic and Sports Medicine Hospital, Doha, Qatar; 2 St George's University of London, Division of Cardiac & Vascular Sciences, London, UK; 3 St Vincent's University Hospital and The Blackrock Clinic, Dublin, Ireland; 4 Royal Brompton and Harefield NHS Trust, London, UK

**Keywords:** Ultra-endurance exercise, hypertrophic cardiomyopathy, athlete's heart, sudden cardiac death, pre-participation screening

## Abstract

Regular and prolonged exercise is associated with increased left ventricular wall thickness that can overlap with hypertrophic cardiomyopathy (HCM). Differentiating physiological from pathological hypertrophy has important implications, since HCM is the commonest cause of exercise-related sudden cardiac death in young individuals. Most deaths have been reported in intermittent 'start-stop' sports such as football (soccer) and basketball. The theory is that individuals with HCM are unable to augment stroke volume sufficiently to meet the demands of endurance sports and are accordingly 'selected-out' of participation in such events. We report the case of an ultra-endurance athlete with 25 years of > 50 km competitive running experience, with genetically confirmed HCM; thereby demonstrating that these can be two compatible entities.

## Background

Regular physical exercise is associated with physiological increases in cardiac dimensions which may be reflected on the electrocardiogram (ECG). Differentiating a physiological or pathological remodelling mechanism is important, as significant cardiac enlargement may be an expression of underlying cardiac disease, placing the athlete at a greater risk of sudden cardiac death (SCD) [1]. Approximately 80% of non-traumatic sudden deaths in young athletes (< 35 years) are caused by inherited or congenital structural and functional cardiovascular abnormalities, which provide a substrate for arrhythmias predisposing to SCD [2]. Hypertrophic cardiomyopathy (HCM), defined by the presence of increased ventricular wall thickness or mass in the absence of loading conditions (hypertension, valve disease, etc) sufficient to cause the observed abnormality [3], is the leading cause of SCD in the young and accounts for one third of all sudden cardiac deaths in young competitive athletes [4,5]. However, existing data also demonstrates that a small proportion of athletes (< 2%) exhibit increased left ventricular wall thickness (LVWT) ranging between 13-16 mm [6-8], which overlaps with morphologically mild HCM.

Deaths from HCM are predominantly confined to intermittent 'start-stop' sports such as American football, basketball and soccer, with few cases reported in endurance sports. The postulated theory is that individuals with HCM are unable to augment cardiac output sufficiently to participate in intensive and prolonged endurance sports due to a combination of pronounced LVH, a non-compliant LV, exercise-induced LV outflow obstruction and microvascular ischemia. However, we report an ultra-endurance athlete with confirmed HCM capable of performing high-levels of aerobic ultra-endurance activity.

## Case Presentation

A 44 year-old Caucasian male was evaluated in our centre for investigation of a cardiac murmur identified by his primary care physician. The individual was asymptomatic with no past medical history, medication history or family history. He was an ultra-marathon runner with over 25 years of competitive running history; currently participating in 3 ultra-marathon (> 50 km) events per year often involving challenging mountainous and frozen terrain. Resting blood pressure of 95/60 mmHg and physical examination was unremarkable apart from the presence of a soft ejection systolic murmur.

**Figure 1:**
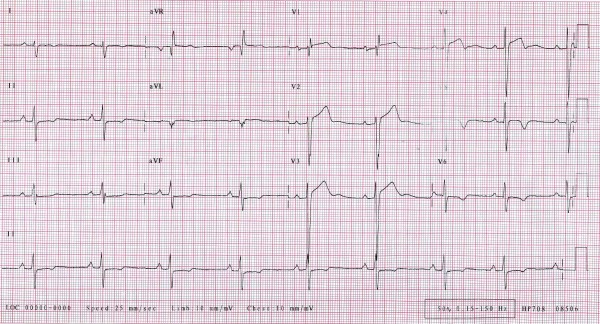
**12-lead ECG of a 44 year old ultra-marathon runner demonstrating first degree heart block, right axis deviation, bi-atrial enlargement, left ventricular hypertrophy with associated ST-segment depression in leads II, III, AVF and deep T-wave inversions in leads V5 and V6**.

**Figure 2:**
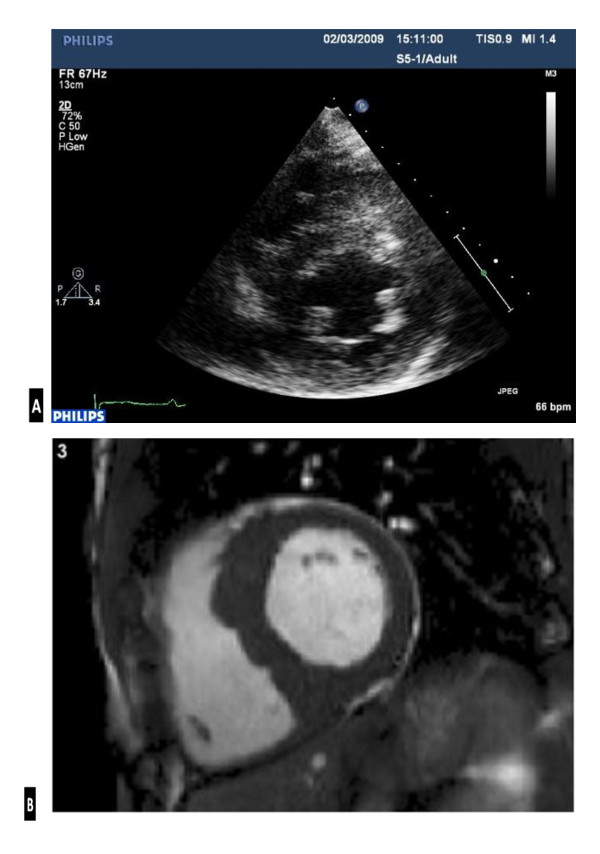
**Trans-thoracic echocardiography (a) vs. CMR (b) image demonstrating; asymmetric septal hypertrophy of 14 mm and a left ventricular cavity size of 44 mm in the parasternal short axis at papillary muscle level (a) vs. asymmetric septal hypertrophy of 17 mm, a left ventricular cavity size of 44 mm and a lateral wall of 8.5 mm at the same level (b)**.

**Figure 3:**
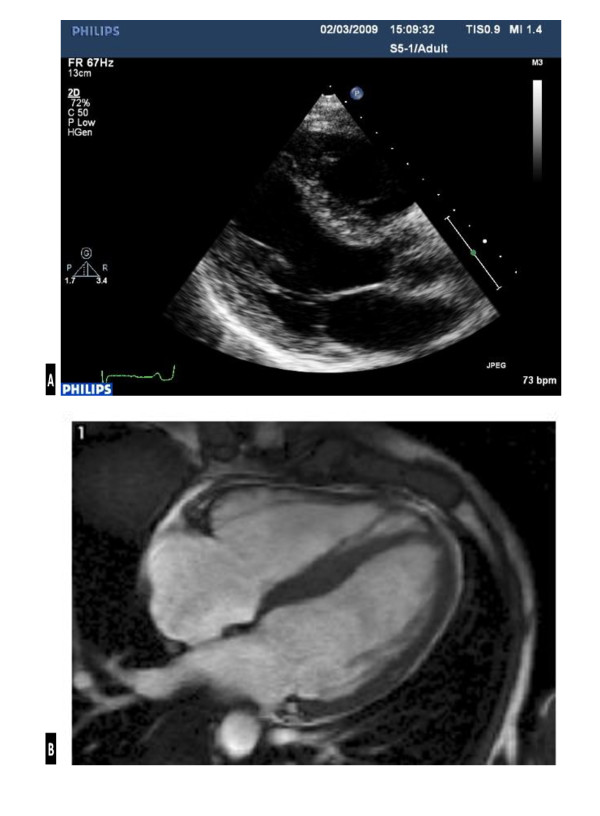
**Trans-thoracic echocardiography (a) vs. CMR (b) image demonstrating; asymmetric septal hypertrophy of 14 mm and a left ventricular cavity size of 44 mm in the parasternal long axis at papillary muscle level (a) vs. asymmetric septal hypertrophy of 17 mm, a left ventricular cavity size of 44 mm and a lateral wall of 8.5 mm at the same level (b)**.

**Figure 4:**
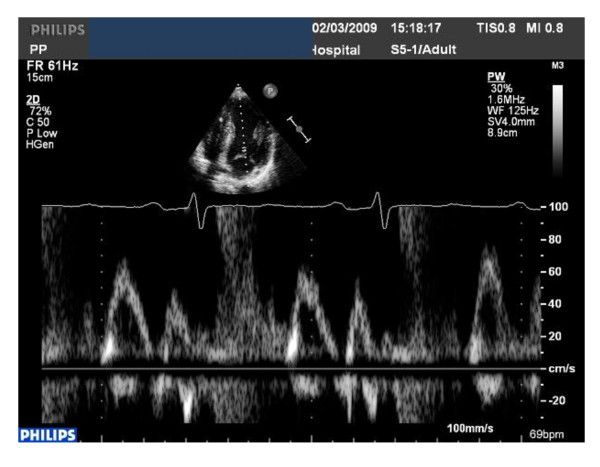
**Normal indices of diastolic function; mitral inflow E:A ratio of > 1**.

**Figure 5:**
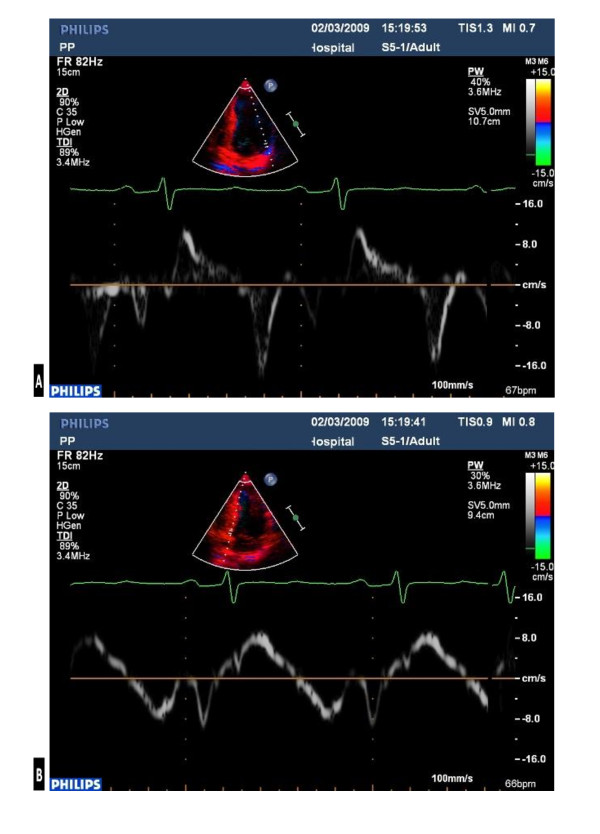
**Tissue Doppler analysis of longitudinal function; with an E' of 16 cm/s in the lateral LV wall (a) and 11 cm/s in the septal LV wall (b)**.

The ECG demonstrated first-degree heart block, right axis deviation, voltage criteria for bi-atrial enlargement, LVH and significant repolarisation anomalies including ST-segment depression in leads II, III and AVF, and deep T-wave inversions in leads V5 and V6 (Figure 1). Echocardiography demonstrated asymmetric septal hypertrophy of the basal and mid-septum with a maximal LVWT of 14 mm and an end-diastolic LV diameter of 44 mm (Figure 2a, 3a and 2b, 3b). There was no evidence of systolic anterior motion of the mitral valve leaflet or LV outflow tract obstruction. Systolic and diastolic function were clinically normal; the left atrial diameter measured 37 mm, the E/A ratio was > 1 (Figure 4) and tissue Doppler revealed an E' of 16 cm/s at the lateral LV wall and 11 cm/s in the septal LV wall (Figure 5).

Subsequent investigations included an exercise stress test with the athlete completing 21 minutes of the Bruce protocol (19.1 METS) corresponding to an oxygen consumption of 67 ml/kg^-1^/min^-1^. Heart rate (91% predicted maximum) and BP response (systolic BP rising from 98 mmHg to 168 mmHg at peak exertion) to exercise was normal and there was no evidence of cardiac dysrhythmias on exercise or on the 24-hour Holter monitor.

**Figure 6:**
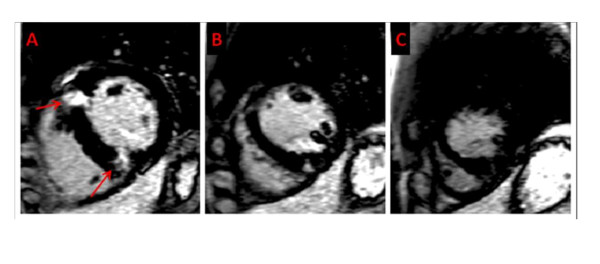
**Late gadolinium enhancement (A-C) at basal (A), mid (B), and apical(C) ventricular levels, demonstrating focal myocardial fibrosis (red arrows) predominantly at basal level in the basal anteroseptal and inferoseptal walls**.

The abnormal resting ECG, asymmetric septal hypertrophy and non-dilated LV cavity raised suspicion of HCM. However, the normal indices of diastolic function and supra-normal functional capacity favoured 'athlete's heart'. Consequently, CMR was performed using standardised imaging protocols [9]. Analysis of the short axis images in diastole demonstrated asymmetrical hypertrophy predominantly affecting the basal and mid anteroseptal and inferoseptal walls (maximum wall thickness, 17 mm). The lateral wall at the same level measured 8.5 mm (Figure 2b, 3b). Imaging for late gadolinium enhancement (LGE) was performed approximately 10 minutes after contrast administration using an inversion-recovery gradient echo sequence. This demonstrated regions of focal intramyocardial fibrosis in the anterior and inferior basal LV-RV insertion points (Figure 6). The degree of focal fibrosis was felt to be disproportionate and not physiological. Insertion point fibrosis has been described in cases of hypertensive LVH, aortic stenosis and congenital heart disease [10]. In this case however, there was no history of any of the above conditions and hence LGE was considered to represent cardiomyopathy.

Accordingly, first-degree relatives were invited to our centre for cardiovascular screening, which demonstrated an abnormal ECG and echocardiogram consistent with HCM in the mother and sister of the index case. Genetic testing for known mutations encoding sarcomeric contractile proteins proved positive for a mutation in the MYBPC3 gene encoding myosin binding protein C (specifically c.2096delC (p.Pro699fs) in MYBPC3 exon 22) in all three individuals.

## Discussion

Previous cases of endurance athletes with HCM have been reported [11], however, this is the first case of a genetically proven diagnosis in an individual able to perform such high levels of ultra-endurance exercise for over 25 continuous years. This case demonstrates the challenges faced when evaluating athletes with an abnormal ECG or LV hypertrophy on echocardiography and highlights the importance of systematic evaluation that includes LGE-CMR to determine whether such changes represent physiological adaptation or pathological phenomena.

The ECG presented here has numerous features compatible with cardiac adaptation to exercise, including voltage criteria for LVH, ST-segment depression, a prolonged PR interval and inverted T-waves [6,12-17]. However, T-wave inversion in V5-6 are nearly always associated with cardiomyopathy and should always be interpreted with suspicion for three important reasons; 1) the ECG is abnormal in 95-97% of patients with HCM, 2) whereas voltage criteria for LVH are present in around 75% of patients with HCM, isolated Sokolow-Lyon voltage criterion for LVH commonly observed within athletes (without associated ST and T wave changes) occurs in only 2% of HCM patients, and 3) repolarisation changes consisting of ST segment shift and T wave inversion are present in over 90% of cases.

Whilst echocardiography demonstrated a LVWT is 14 mm at the septum, it is well established that a minority of Caucasian athletes (< 2%) also demonstrate physiological LVH between 13-16 mm [7,8,18]. However, physiological LVH is typically associated with LV cavity dilatation of 55-65 mm. Hence, the LV cavity size of 44 mm in this case is unexpectedly reduced and typical of the disparity seen in individuals with HCM. Given the diagnostic uncertainty in this athlete, this case study also highlights the important role of including CMR in the workup of individuals presenting "grey zone" LVH (12-15 mm). There was a major discrepancy between maximal LV wall thickness derived by echocardiography (14 mm) and that of CMR (17 mm). Indeed, a wall thickness of 17 mm is not routinely observed within athletes regardless of body surface area [7,8] and points ominously towards pathology. CMR provides a comprehensive assessment of both ischemic and non-ischemic cardiomyopathies providing detailed precise information on cardiac anatomy, function, tissue characterisation, epicardial and microvascular perfusion, valvular flows, and coronary and peripheral angiography. Measurements of maximal wall thickness are highly accurate, as is the pattern definition of LV wall thickening (focal vs. mild concentric) and unlike echocardiography, no geometrical assumptions need to be made about the ventricle [19,20]. Indeed, in some regions of the LV chamber, the extent of hypertrophy can be underestimated by echocardiography compared to CMR [21,22], which is not diagnostically helpful in "grey zone" athletes. Finally, LGE provides a sensitive tool for the detection of myocardial fibrosis, abnormalities not typically seen in physiological LVH, thus highlighting pathology [23-25].

Due to the abnormal CMR and the abnormal cardiovascular evaluation of first-degree relatives, familial disease was presumed and genetic testing confirmed a diagnosis of HCM. Variable expression of the disease is common, even amongst members of the same family sharing the same gene defect. Mutations in more than 13 genes encoding sarcomeric contractile proteins have been identified as a cause of HCM [26]. Importantly, in a patient with an overt cardiomyopathy, the yield or rate of mutation identification is variable (50-70%) [27]. Failure to identify a recognised mutation does not exclude the diagnosis of a cardiomyopathy for three important reasons; 1) not all genetic regions are assessed, 2) current technology is not able to detect some forms of mutation (intronic cryptic splice sites, large genomic rearrangements, etc), and 3) a similar phenotype may possibly develop without a specific genetic constitution. Lastly, it must be taken into account that genetic testing is expensive, not routinely available in most cardiology departments and can take up to 9 months to get a result. In our opinion, whilst the genetic test confirmed HCM, it was the abnormal electrocardiographic, imaging findings and family screening that confirmed genetic HCM rather than an athlete's heart. The clinical impression was that if a causative gene for HCM was not identified, the diagnosis of HCM would still have been made.

Recently, our laboratory recently examined the cardiac structure and function of a unique cohort of 12 asymptomatic truly life-long, competitive veteran endurance athletes (56 ± 6 yr), with 20 age-matched veteran controls (60 ± 5 yr) and 17 younger male endurance athletes (31 ± 5 yr) using LGE CMR [28]. Veteran athletes had a significantly larger LV and RV end-diastolic and systolic volumes, intraventricular septum thickness during diastole [mean ± SD; (range) 11 ± 1 mm (9-13 mm) vs. 10 ± 2 mm (10-13 mm), p < 0.05] and posterior wall thickness during diastole [10 ± 1 mm (8 - 11 mm) vs. 8 ± 1 mm (7 - 10 mm) p < 0.001], together with significantly reduced LV and RV ejection fractions (p *<*0.05), compared with veteran controls. We also observed the presence of myocardial fibrosis in 6 (50%) of the veteran athletes, but no LGE in the age-matched veteran controls or young athletes. Importantly, the prevalence of LGE in veteran athletes was significantly associated with the number of years spent training (p < 0.001), number of competitive marathons (p < 0.001) and ultra-endurance (> 50 miles) marathons (p < 0.007) completed, suggesting a link between life-long endurance exercise and 'acquired' myocardial fibrosis that requires further investigation. However, the extent of LVH and fibrosis observed with these life-long veteran athletes was not as extensive as the athlete presented here, thus pointing towards a pathological mechanism.

Pre-participation screening data from Italy, incorporating the 12-lead ECG, suggests that the incidence of sudden death from HCM may be reduced through earlier identification and subsequent disqualification of affected athletes from competitive sport [29]. Guidelines from both the ACC 36th Bethesda Conference and ESC recommend that athletes with unequivocal or 'probable' HCM abstain from competitive sport and vigorous training with the exception of low-intensity activities [30-32]. The pathophysiology of death in an athlete with HCM during sport is multi-factorial; HCM has distinctive histology with affected areas of the myocardium demonstrating considerable interstitial fibrosis with gross disorganisation of the muscle bundles, resulting in a characteristic whorled pattern. In patients with HCM, the presence of fibrosis is an important marker of risk and patients with a greater number of risk factors for SCD typically have more fibrosis (as found in post-mortem data) [33], that is an independent risk for major adverse cardiac events [34]. The presence of fibrosis contributes to the disruption of the electrical synchrony that exists between myocytes and thereby increases arrhythmic potential [35,36]. Although a risk stratification algorithm for HCM is in existence, extrapolation of such data to an athletic milieu with associated high circulating catecholamines, acid-base shifts and electrolyte imbalances is unrealistic. Based on these considerations the exercise guidelines for this heterogeneous disorder are homogenous and conservative [37], and include athletes who may genuinely be at low risk of fatal cardiac events, as in this particular case.

Sporting disqualification from all high-intensity ultra-endurance activity was discussed with the athlete together with ICD insertion. Personal and family genetic counselling together with clinical symptom education was given to the athlete regarding the risks of ultra-endurance exercise and the potential for SCD. The athlete and family members agreed for genetic testing, but the athlete refused an ICD. Accordingly, the athlete is required to undergo a comprehensive yearly cardiovascular examination. However, the athlete continues to compete in ultra-endurance running events despite knowing the risks posed by continued high intensity competition; and with 2 years of follow-up data he remains asymptomatic without any significant cardiac changes.

## Conclusion

**Figure 7:**
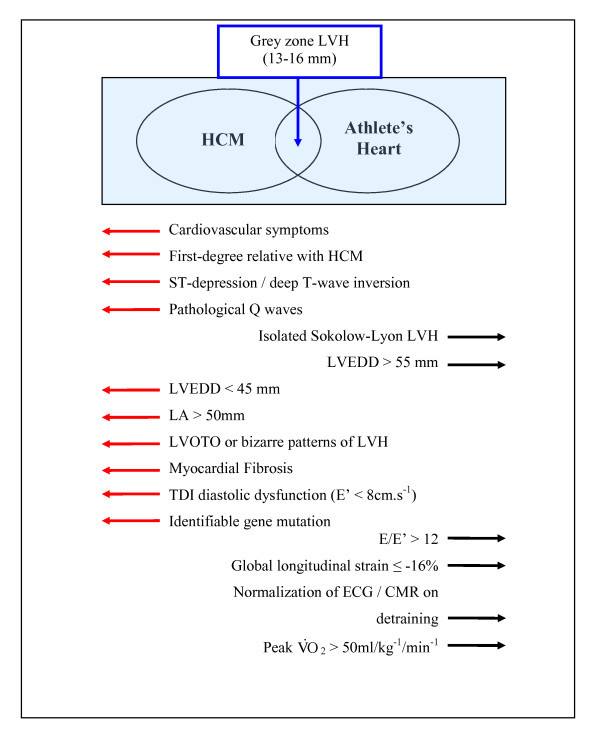
**Clinical criteria used to help differentiate athlete's heart from HCM in cases with a borderline degree of LVH (13-16 mm)**. LVH = left ventricular hypertrophy; HCM = hypertrophic cardiomyopathy; BSA = body surface area; LVWT = left ventricular wall thickness; LVEDD = left ventricular end-diastolic diameter; LVOTO = left ventricular outflow tract obstruction; V02 = oxygen consumption; TDI = tissue doppler imaging.

This case study reports an asymptomatic male athlete with 25 years of ultra-endurance competition, with genetically confirmed HCM phenotypically manifesting with LVH, a small LV cavity together with repolarisation abnormalities suggestive of HCM. Despite documented asymmetric hypertrophy and focal myocardial fibrosis in the basal anteroseptal and inferoseptal walls, it is suspected that the athlete is able to run ultra-marathons due to a compliant LV with normal diastolic and systolic parameters, which is able to augment stroke volume. In conclusion, rare as they might be, a minority of HCM patients are capable of life-long careers in ultra-endurance exercise. This case also highlights the importance of systematic evaluation of all athletes with electrocardiographic features suggestive of a cardiomyopathy or ion channelopathy, with LGE CMR (Figure 7), maximal cardiopulmonary stress testing, first-degree family screening and where appropriate, genetic testing, to determine whether such changes represent physiological adaptation or pathological phenomena.

## Consent

Written informed consent was obtained from the patient for publication of this case report and any accompanying images. A copy of the written consent is available for review by the Editor-in-Chief of this journal.

## Abbreviations

HCM: Hypertrophic cardiomyopathy; SCD: Sudden cardiac death; LV: Left ventricle; LVH: Left ventricular hypertrophy; LVWT: Left ventricular wall thickness; CMR: Cardiac magnetic resonance.

## Competing interests

The authors declare that they have no competing interests.

## Authors' contributions

MGW, NC and SS designed the case, MGW, NC, MP, ROH and SS collected and analyzed the data, MGW and NC wrote the preliminary draft of the manuscript and all authors supplied comments and corrections, SS is the guarantor. All authors read and approved the final manuscript.

## Funding

Authors NC and MP are funded by a research grant from the charitable organisation 'Cardiac Risk in the Young' (Epson Downs, United Kingdom). SKP was supported by the NIHR Cardiovascular Biomedical Research Unit of the Royal Brompton and Harefield NHS Foundation Trust and Imperial College.
